# Molecular Characteristics and Epidemiology of Meningococcal Carriage, Burkina Faso, 2003

**DOI:** 10.3201/eid1306.061395

**Published:** 2007-06

**Authors:** Judith E. Mueller, Lassana Sangaré, Berthe-Marie Njanpop-Lafourcade, Zekiba Tarnagda, Yves Traoré, Seydou Yaro, Raymond Borrow, Bradford D. Gessner, Pierre Nicolas

**Affiliations:** * Agence de Médecine Préventive, Paris, France; † Centre Hospitalier Universitaire Yalgado Ouédraogo, Ouagadougou, Burkina Faso; ‡ Institut de Recherche en Science de la Santé, Bobo-Dioulasso, Burkina Faso; § Université de Ouagadougou, Ouagadougou, Burkina Faso; ¶ Centre Muraz, Bobo-Dioulasso, Burkina Faso; # Health Protection Agency, Manchester, United Kingdom; ** Institut de Médecine Tropicale du Service de Santé des Arnées, Marseille, France

**Keywords:** Neisseria meningitidis, carriage, Africa, ST-11, serogroup W135, serogroup A, Burkina Faso, research

## Abstract

To describe *Neisseria meningitidis* strains in the African meningitis belt in 2003, we obtained 2,389 oropharyngeal swabs at 5 monthly visits in a representative population sample (age range 4–29 years) in Bobo-Dioulasso, Burkina Faso. A total of 152 carriage isolates were grouped, serotyped, and genotyped. Most isolates were NG:NT:NST sequence type (ST) 192 (63% of all *N. meningitidis*), followed by W135:2a:P1.5,2 of ST-11 (16%) and NG:15:P1.6 of ST-198 (12%). We also found ST-2881 (W135:NT:P1.5,2), ST-751 (X:NT:P1.5), and ST-4375 (Y:14:P1.5,2) but not serogroups A or C. Estimated average duration of carriage was 30 days (95% confidence interval 24–36 days). In the context of endemic group W135 and meningococcal A disease, we found substantial diversity in strains carried, including all strains currently involved in meningitis in this population, except for serogroup A. These findings show the need for large samples and a longitudinal design for *N. meningitidis* serogroup A carriage studies.

## Abstract 2

Meningococcal serogroups are genetically diverse and short-lived in the African meningitis belt.

In the African meningitis belt, serogroup A of *Neisseria meningitidis* (NmA) is the most frequent cause of bacterial meningitis. Since an epidemic in 1987, these invasive NmA strains have been identified as belonging to clonal complex sequence type (ST)–5 (*1*). From 1980 through 2000, meningococcal serogroup W135 (NmW135) was found in Africa only occasionally (*2*) and never as an epidemic strain. At the end of the 2001 epidemic season in Burkina Faso and Niger, however, similar proportions of cases caused by NmW135 and NmA were found (*3*). During a large epidemic in Burkina Faso in 2002, phenotype W135:2a:P1.5,2 was the predominant strain; this strain belonged to the ST-11 clonal complex (*4*), as did NmW135 strains found in an outbreak among Hajj pilgrims in 2000. Since early 2003, NmW135 has gradually decreased, and in 2005 and 2006 NmA again predominated, with some NmW135 outbreaks in Uganda, Sudan, and Kenya (*5*,*6*).

Most published carriage studies on sub-Saharan Africa were conducted after outbreaks, with transversal design or with nonsystematic specimen collection. These studies often found a predominance of the outbreak strain. Our longitudinal study describes meningococcal phenotypes and genotypes circulating in an urban Burkina Faso population 1 year after an *N*. *meningitidis* W135 epidemic, their dynamics during a nonepidemic meningitis season, and the carriage prevalence of disease-causing strains in the healthy population.

## Methods

### Recruitment and Swab Collection

Methods, population characteristics, and 4-month carriage prevalence by serogroup have been previously reported (*7*). The study was reviewed and approved by the Ethics Committee of Centre Muraz, Bobo-Dioulasso, Burkina Faso, and the Comité de Vigilance of Pasteur Institute, Paris. Briefly, after written informed consent was obtained from study participants or their guardians (for persons <18 years of age), a random sample of the healthy residents of urban Bobo-Dioulasso, Burkina Faso, were examined at 5 clinic visits from February 3 to June 7, 2003. The sampling design required that 1 participant 4–14 years of age and 1 participant 15–29 years of age were included from each selected compound (community of households). At all 5 visits, swabs were taken from the posterior pharyngeal wall through the mouth by using cotton-tipped sterile swabs, which were streaked immediately onto plates containing selective medium. The plates were stored immediately in an atmosphere of 5% CO_2_ at room temperature for a maximum of 2 hours until incubation at 37°C.

### Microbiologic Analyses

*N*. *meningitidis* strains from incubated plates were isolated and identified by using established bacteriologic methods, following recommendations of the World Health Organization when applicable (*8*). *N*. *lactamica* isolates were also cultured and identified. Confirmation and genogroup prediction of *N*. *meningitidis* isolates was conducted on the basis of PCR testing as previously described (*9*,*10*). All groupable *N*. *meningitidis* isolates and a subset of nongroupable isolates were further tested with immune serum for serogroup confirmation.

Serotypes and serosubtypes were determined by using monoclonal antibody kits obtained from the National Institute of Public Health and the Environment (Bilthoven, the Netherlands) by the whole-cell enzyme immunoassay technique, as previously described (*11*). Chromosomal DNA restriction patterns were analyzed by pulsed-field gel electrophoresis (PFGE). Whole chromosome DNA macrorestriction fragments generated by digestion with *Spe*I endonuclease were separated by PFGE as previously described (*12*). DNA fragments were separated by using a Chef-DR II system (Bio-Rad Laboratories, Hercules, CA, USA). PFGE fingerprint patterns were compared by using the criteria of Tenover et al. (*13*).

**Figure 1:**
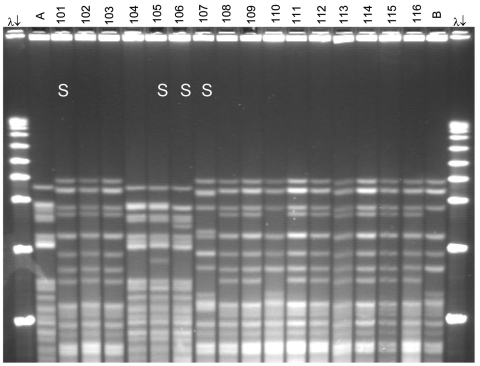
Pulsed-field gel electrophoresis (PFGE) analysis of chromosomal DNA from pharyngeal meningococcus isolates (stained with ethidium bromide). Whole chromosome DNA macrorestriction fragments were generated by digestion with endonuclease *Spe*I. Shown are examples of sequence type (ST) prediction by PFGE in carried meningococci and diversity among STs. S, isolates tested by multilocus sequence typing (MLST). Lanes λ (arrows), PFGE marker I (Boehringer Mannheim, Mannheim, Germany); lane A, ST-2881, meningitis case isolate, Niger 2003; lanes 101, 102, and 103, ST-11, W135:2a:P1.5,2; lanes 104 and 105, ST-2881, W135:NT:P1.5,2; lane 106, ST-4151, W135:NT:P1.5,2; lane 107, ST-11_2000_, W135:NT:P1.5,2; lanes 108–116, ST-11, W135:2a:P1.5,2; lane B, meningitis case isolate, ST-11, Niger 2003. Isolates 101 and 107 were identified as ST-11 by MLST. Isolates 102, 103, 108, 109, and 111–116 are indistinguishable from isolate 101 and are therefore considered ST-11. The 19 ST-11 isolates had 4 different PFGE patterns, of which 3 are represented by isolates 101, 107, and 110. The pattern of isolate 107 is indistinguishable from the 2000 Hajj epidemic strain (not shown).

**Figure 2:**
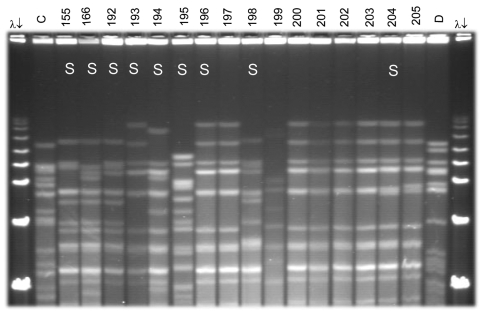
Pulsed-field gel electrophoresis (PFGE) analysis of chromosomal DNA from pharyngeal meningococcus isolates (stained with ethidium bromide). Whole chromosome DNA macrorestriction fragments were generated by digestion with endonuclease *Spe*I. Shown are examples of sequence type (ST) prediction by PFGE in carried meningococci and diversity among STs types. S, isolates tested by multilocus sequence typing (MLST). Lanes λ (arrows) PFGE marker I (Boehringer Mannheim, Mannheim, Germany); lane C, ST-2881, meningitis case isolate, Niger 2003; lanes 155, 166, and 192–194, ST-192, NG:NT:NST; lane 195, ST-198, NG:15:P1.6; lanes 196–198, ST-192, NG:NT:NST; lane 199, isolate unrelated to the presented study; lanes 200–205, ST-192, NG:NT:NST; lane D, meningitis case isolate, ST-11, Niger 2003. Isolates 192, 193, 194, 196, 198, and 204 were identified as ST-192 by MLST. Isolates 197, 200, 201, 202, and 203 are indistinguishable from isolate 196 and are thus considered ST-192. ST-192 isolates from this study had 10 different PFGE patterns, of which 6 are represented by isolates 155, 166, 192, 194, 196, and 204.

Multilocus sequence typing (MLST) was performed on a subset of 53 isolates chosen to represent different PFGE variants of different serogroups (*14*). Fragments from 7 housekeeping genes (*abc*Z, *adk*, *aro*E, *fum*C, *gdh*, *pdh*C, and *pgm*) were used for typing, as given on the *Neisseria* MLST website (http://pubmlst.org/neisseria/). After DNA preparation and amplification by PCR, each locus sequence was analyzed on an ABI Prism 3100 DNA sequencer (Applied Biosystems, Foster City, CA, USA). Sequence analysis was performed by using Vector NTI suite software (InforMax, Bethesda, MD, USA). The sequences were compared with existing alleles on the *Neisseria* MLST website for determination of allele numbers, STs, and clonal complexes of the isolate. After internal validation tests, strains that had the same PFGE profile were considered to belong to the same ST, and thus were defined as having the same ST (Figures 1, 2).

### Estimation of Carriage Duration

Mean duration of carriage and 95% confidence intervals (CIs) were calculated by the truncated observations method described by de Wals and Bouckaert (*15*). We assumed that isolates with identical phenotypes and genotypes collected from a person at consecutive visits, and only those, indicated an ongoing carriage event.

## Results

A total of 488 persons were included in the study; >96% were seen at each respective visit. Eighteen percent of the population carried a meningococcus at least once during the study. All genogroupable isolates could be serogrouped. The 152 meningococcal isolates were attributed to serogroups NmW135 (n = 28), NmX (n = 5), NmY (n = 3), and nongroupable, autoagglutinable, or polyagglutinable Nm (n = 116). No NmA, NmB, or NmC were found (Table 1).

**Table 1:** Characterization of 152 meningococcal isolates, Bobo-Dioulasso, Burkina Faso, 2003*

Sequence type (ST)	No. isolates (% ST)	No. tested by MLST	Phenotype	No. (%) ST isolates with phenotype
ST-192	96 (63)	40	NG:NT:NST	95 (63)
			W135:NT:NST	1 (1)
ST-11	19 (13)	5	W135:2a:P1.5,2	16 (11)
			W135:NT:P1.5,2	2 (1)
			NG:2a:P1.5,2	1 (1)
ST-198	13 (9)	3	NG:15:P1.6	12 (8)
			W135:15:P1.6	1 (1)
ST-4426 (clonal complex ST-198)	2 (1)	2	NG:15:P1.6	2 (1)
ST-2881	8 (5)	2	W135:NT:P1.5,2	8 (5)
ST-4151 (single locus variant of ST-2881)	1 (1)	1	W135:NT:P1.5,2	1 (1)
ST-751	5 (3)	3	X:NT:P1.5	5 (3)
ST-4376 (single locus variant of ST-751)	1 (1)	1	NG:NT:P1.5	1 (1)
ST-4375 (clonal complex ST-23)	3 (2)	1	Y:14:P1.5,2	3 (2)
ST-2049	1 (1)	1	NG:15:P1.6	1 (1)
ST-4377	2 (1)	2	NG:NT:NST	2 (1)
Not tested	1 (1)	–	NG: Not determined	1 (1)
Total	152 (100)	61		152 (100)

*MLST, multilocus sequence typing. NG includes nongroupable, autoagglutinable, and polyagglutinable strains.

**Figure 3:**
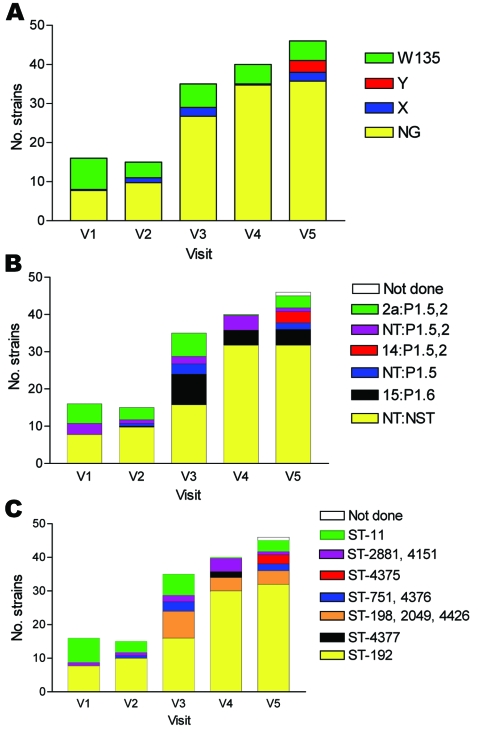
Serogroups (A), serotypes (B), and sequence types (C) of 152 meningococci carried by 488 persons obtained at 5 monthly study visits from February through June 2003, Bobo-Dioulasso, Burkina Faso. NG, nongroupable; ST, sequence type.

Among the 151 meningococcal isolates submitted for serotyping, most could not be serotyped or serosubtyped with existing antibodies (n = 98, 65%). Serotype 2a:P1.5,2 (n = 17, 11%) and 15:P1.6 (n = 16, 11%) were the most frequently found serotypes, followed by NT:P1.5,2 (n = 11, 7%), NT:P1.5 (n = 6, 4%), and 14:P1.5,2 (n = 3, 2%) (Figure 3, Table 1).

Among the 151 isolates analyzed by MLST or PFGE, most were ST-192 (n = 96, 63%), followed by ST-11 (n = 19, 13%), ST-198 (n = 13, 9%), and ST-2881 (n = 8, 6%, including 1 strain with the single locus variant ST-4151) (Table 1). Other STs represented <5% of the carriage strains and included ST-4426 (in the clonal complex ST-198), ST-751 (including the single locus variant ST-4376), ST-4375 (in the clonal complex ST-23), and ST-4377.

Serogroup W135 was mostly found in combination with serotype 2a:P1.5,2 and genotype ST-11. Serogroup X was found with NT:P1.5, ST-751, and serogroup Y was found with 14:P1.5,2, ST-4375 (Table 1).

### Diversity among STs

The 19 isolates belonging to ST-11 showed moderate diversity in restriction patterns (Figure 1). Three (16%) isolates found in February 2003, among them 2 isolates with phenotype W135:NT:P1.5,2, were indistinguishable from the 2000 Hajj outbreak strain, and 1 (5%) isolate each showed 1- and 2-band differences from the 2000 Hajj strain. The other 14 ST-11 isolates (74%), among them an isolate with phenotype NG:2a:P1.5,2, showed a 6-band difference from the 2000 Hajj strain.

The 8 ST-2881 isolates belonged to 1 clone with a 0- to 2-band difference between them. Although most of the ST-2881 isolates belonged to group W135, their PFGE patterns were unrelated to ST-11 isolates in group W135; they were closely related to ST-2881 invasive strains of serogroup W135 found in 2003 in Niger (*16*).

Among the 96 ST-192 isolates, 86 had interpretable results by PFGE, which showed considerable diversity in restriction patterns (Figure 2). Of these isolates, 41 (48%) were indistinguishable from each other and 10 (12%), among them the isolate with phenotype W135:NT:NST, were closely related with 1- to 3-band differences. Thirty-one isolates (36%) were possibly related to the central clone (4- to 6-band difference), and 4 (5%) were unrelated (>7-band difference).

### Carriage Dynamics

The gradual increase in carriage point prevalence of any Nm from 3.5% in February to 9.9% in May–June was caused by an increase in ST-192 with a phenotype of NG:NT:NST (Figure 3). The number of isolates of this genotype and phenotype at each of the 5 visits was 7, 8, 15, 26, and 27, respectively.

Among the 84 persons that carried meningococci during >1 visit, half (n = 42) were carriers at only 1 visit and half at multiple visits. Of the 42 persons with meningococcal carriage at several visits, 21 always had the same strain (Table 2) and 19 had different strains (Table 3). The latter group carried <3 different strains during the 4 months of observation, and 4 persons had the same strain on multiple occasions but with interruption. Twenty-seven persons (32% of all carriers) had the same strain >2 times at subsequent visits. The mean duration of carriage for all serogroups was estimated as 30 days (95% CI 24–36 days). Estimated mean carriage duration was 20 days (95% CI 15–23 days) for NmW135 and 34 days (95% CI 27–42 days) for nongroupable strains. The estimated mean duration of overall Nm carriage increased with the increasing prevalence of nongroupable strains toward the end of the meningitis season: 21 days (95% CI 18–24 days) during February–April compared with 35 days (95% CI 28–43 days) during April–June.

**Table 2:** Analysis of 21 persons carrying the same meningococcal strain at 5 monthly visits, Bobo-Dioulasso, Burkina Faso, 2003

Person	Age, y	Visit 1	Visit 2	Visit 3	Visit 4	Visit 5
1	4	W135: ST-2881		W135: ST-2881		
2	6			NG:ST-192	NG:ST-192	NG:ST-192
3	7	NG:ST-192			NG:ST-192	NG:ST-192
4	8	NG:ST-192			NG:ST-192	
5	10			NG:ST-192	NG:ST-192	NG:ST-192
6	11				NG:ST-192	NG:ST-192
7	11				NG:ST-192	NG:ST-192
8	12			NG:ST-192	NG:ST-192	NG:ST-192
9	14			NG:ST-192	NG:ST-192	NG:ST-192
10	14		NG:ST-192		NG:ST-192	
11	14			NG:ST-192	NG:ST-192	NG:ST-192
12	15		NG:ST-192			NG:ST-192
13	16				NG:ST-198	NG:ST-198
14	17				NG:ST-192	NG:ST-192
15	17			NG:ST-192	NG:ST-192	
16	18			NG:ST-192	NG:ST-192	NG:ST-192
17	19		NG:ST-192	NG:ST-192		NG:ST-192
18	20	NG:ST-192			NG:ST-192	NG:ST-192
19	20				NG:ST-192	NG:ST-192
20	22		NG:ST-192			NG:ST-192
21	26		W135: ST-11			W135: ST-11

**Table 3:** Analysis of 19 persons carrying >1 meningococcal strain at 5 monthly visits, Bobo-Dioulasso, Burkina Faso, 2003

Person	Age, y	Visit 1	Visit 2	Visit 3	Visit 4	Visit 5
22	4			NG: ST-198	NG:ST-192	NG:ST-192
23	5	W135: ST-11			NG:ST-192	NG:ST-192
24	8				W135: ST-2881	NG:ST-4426
25	8			X: ST-751	NG:ST-192	X: ST-751
26	9		NG:ST-192	W135: ST-11		
27	11			W135: ST-11	NG:ST-192	W135: ST-2881
28	13			NG: ST-198	NG:ST-192	NG:ST-192
29	14			NG: ST-198	NG:ST-192	
30	16				W135: ST-2881	Y:ST-4375
31	18		W135: ST-11	NG:ST-2049	NG: ST-198	NG: ST-198
32	18			NG:ST-11	W135: ST-2881	NG:ST-192
33	18		NG:ST-192	NG: ST-198	NG:ST-192	
34	20			NG: ST-198	NG: ST-192	NG: ST-192
35	21	W135: ST-11		NG:ST-192	W135: ST-11	
36	22		X: ST-751	NG: ST-198	NG:ST-192	X: ST-751
37	22			X: ST-751	W135: ST-2881	
38	23			W135: ST-11	NG:ST-4377	NG:ST-192
39	26		NG:ST-192		NG:ST-4377	
40	26				NG: ST-192	NG: ST-4426

In addition to the 152 meningococci, 103 *N*. *lactamica* were isolated. Prevalence of *N*. *lactamica* was highest in 4 to 8-year-old children and increased gradually from 5.5% (95% CI 2.1%–13.2%) at the first visit to 16.1% (95% CI 10.9%–23.1%) at the last visit. For persons 9–18 years of age, prevalence of *N*. *lactamica* carriage varied from 2% to 4% over the 5 visits; carriage for adults was >1.5%.

## Discussion

This longitudinal carriage study in a healthy young population in the African meningitis belt describes the diversity of carried meningococcal serogroups, serotypes, and genotypes during a nonepidemic meningitis season. Eleven STs and 4 serogroup categories (including nongroupable strains) were identified. Parallel culture- and PCR-based meningitis surveillance in this population during 2003 showed a high incidence of endemic meningococcal disease (annual rate = 77/100,000 among persons <5 years of age and 5/100,000 among persons >14 years of age). During February–April 2003, 9 cases of NmA meningitis and 28 cases of NmW135 meningitis were found in urban Bobo-Dioulasso (435,000 inhabitants), as well as sporadic cases caused by serogroup X and nongroupable meningococci (Table 4) (*5*,*17*).

**Table 4:** Meningococci isolated during surveillance of acute bacterial meningitis, Bobo-Dioulasso, Burkina Faso, February–April 2003

Sequence type (ST)	Phenotype	No. cases
ST-11	W135:2a:P1.5,2	28
ST-2859 (ST-5 complex)	A:4:P1.9	9
ST-751	X:NT:P1.5	1
ST-192	NG:NT:NST	2

Despite frequent serogroup A disease, no serogroup A meningococcal carriage was found. NmA was likely circulating at low levels during our study but not found because of low transmission density or short duration of NmA carriage, together with sample size limitation. Our study thus provides evidence for low prevalence of serogroup A carriage in nonepidemic conditions, which is similar to results of a study in Nigerian schoolchildren (*18*). This finding is useful for assessment of group A conjugate meningococcal vaccines by carriage studies. To show a reduction in NmA carriage prevalence after vaccination, as was recently reported from the United Kingdom for group C conjugate vaccine (*19*), studies need to include several thousand persons to achieve appropriate statistical power.

Apart from NmA, all phenotypes and genotypes isolated from meningococcal meningitis cases in this population from 2000 to 2005 (Table 5) were represented in this 4-month carriage study of 488 persons. This finding supports the use of carriage studies in nonepidemic conditions for surveillance of meningococcal strains of specific serogroups. For surveillance of new genotypes expressing a group A capsule, however, disease surveillance will be more appropriate. For example, ST-2859, a new genotype that expresses group A capsule, has become a major meningitis agent in Bobo-Dioulasso since 2002 (*17*). Our carriage study did not detect this development. In addition, results from localized carriage studies should not be generalized to West Africa and the African meningitis belt as a whole because only 4 of 7 serogroups and 5 of 15 genotypes found in meningococcal meningitis cases in the region during 2000–2005 were represented in our carriage study.

**Table 5:** Overview of meningococci reported from meningitis patients in West Africa and the African meningitis belt, 2000–2005*

Sequence type (ST)	Phenotype	Place and time of meningococcal disease cases
ST-11	W135:2a:P1.5,2	Sporadic in Cameroon, Senegal, Burkina Faso, Central African Republic, Chad, Niger, and Ghana since 2003. Epidemic and major seasonal agent in Burkina Faso during 2002–2004 (including Bobo-Dioulasso)
	W135:NT:P1.5,2	Sporadic in Bobo-Dioulasso, 2004
	W135:2a:P1.2, W135:NT:P1.2	Sporadic in Niger, 2003
	Y:14:P1.5,2	Sporadic in Bobo-Dioulasso, 2004
ST-1966 (ST-11 complex)	W135:2a:P1.5,2	Sporadic in Burkina Faso
ST-2881	W135:NT:P1.5,2	Sporadic in Benin since 2003. Major seasonal agent in Niger during 2003
ST-5	A:4:P1.9, A:21:P1.9	Major seasonal agent in Niger, Senegal, and Burkina Faso during 2000–2001
ST-7 (ST-5 complex)	A:4:P1.9, A:21:P1.9	Major seasonal agent and epidemic in Cameroon, Chad, Niger, Senegal, Benin, Burkina Faso, Ethiopia, and Nigeria
ST-2859 (ST-5 complex)	A:4:P1.9, A:21:P1.9	Major seasonal agent and epidemic in Burkina Faso since 2003 (including Bobo-Dioulasso)
ST-751	X:NT:P1.5, X:NT:P1.5,2	Sporadic in Burkina Faso (including Bobo-Dioulasso), Niger, and Ghana
ST-181	X:NT:P1.5	Sporadic in Niger
ST-2880	Y:14:P1.5,2	Sporadic in Niger
ST-4375 (ST-23 complex)	Polyagglutinable:14:P1.5,2	Sporadic in Bobo-Dioulasso, 2004
	W135:NT:P1.5,2	Sporadic in Bobo-Dioulasso, 2004
ST-23 (ST-23 complex)	Y:14:NST	Sporadic in Senegal
ST-32, ST-2496 (ST-32 complex), ST-291 (ST-41/44 complex)	C:4:P1.16, B:4:P1.16, B:4:P1.7,16, B:4:P1.9	Sporadic in Cameroon
ST-192	NG:NT:NST	Sporadic in Bobo-Dioulasso, 2003 and 2004
*Data were obtained from references *1**,**5**,**16**,**17**,**20**–**22*, and the *Neisseria* multilocus sequencing typing website (http://pubmlst.org/ neisseria).		

During bacterial meningitis surveillance in the Bobo-Dioulasso population in 2004, we observed 2 invasive strains whose genotypes had been associated with different serogroups and serotypes in our carriage study 1 year earlier (Table 5). ST-11, which is usually associated with phenotype W135:2a:P1.5,2 in invasive strains, had phenotype Y:14:P1.5,2 (seen in ST-4375 carriage strains), and ST-4375, which is usually associated with phenotype Y:14:P1.5,2, had phenotype W135:NT:P1.5,2 (seen in ST-11 carriage strains). These findings could be evidence for a capsular and serotype switch between co-colonizing meningococci, as described by Swartley et al. (*23*). However, that report described only gene conversion for capsule expression, not for outer membrane protein (PorB) expression. The potential capacity of meningococci to exchange capsular plus subcapsular genes needs to be further evaluated.

Our study and previous studies of meningococci in sub-Saharan Africa have shown a similar number of different serogroup categories, including nonserogroupable strains (*18*,*24*–*26*). However, assessing whether the genetic diversity we found is a new phenomenon is difficult because most studies do not report genotypes of all isolates. Five ST strains expressed group W135 capsule in this population that was followed up over a 4-month period. This variation has not been reported for other meningococcal serogroups in sub-Saharan Africa but is consistent with results of a report on increasing genetic diversity of W135-encapsulated strains in France since the Hajj-associated outbreak in 2000 (*27*). In contrast to NmW135, NmA has a relatively low genetic diversity, with only 6 genotypes found to express the A capsule over the past 30 years ([*1*]; http://pubmlst.org/neisseria). This difference between the 2 serogroups suggests that NmW135 may not replace NmA as the major epidemic agent in the future. Nevertheless, the easy adoption of a W135 capsule by various genotype stains, in combination with infrequent immune induction by NmW135 carriage (*7*), may cause regular NmW135 outbreaks to occur.

Nongroupable strains were predominant in our study and other carriage studies during nonepidemic conditions in Burkina Faso, Ghana, Europe, and the United States (*20**,**24**,**28*–*31*). In our study, nongroupable and nontypeable isolates were predominantly ST-192, which represented 63% of all carried meningococci. Data from the *Neisseria* MLST website indicate that ST-192 isolates were present in The Gambia and Niger in the 1990s, but no published data are available on the dimension of prevalence of this strain in these or other countries. This strain deserves closer observation because in Bobo-Dioulasso during 2003 and 2004, 3 persons were found with disease caused by nongroupable ST-192 isolates. Unencapsulated strains rarely cause invasive disease and usually only among complement-deficient persons (*32*). This may have occurred in the 3 patients, whose complement status was not determined. However, the isolates from Bobo-Dioulasso also showed enhanced capacity to escape human immune defenses (*33*), which would enable these isolates to cause invasive disease in immunocompetent persons.

**Figure 4:**
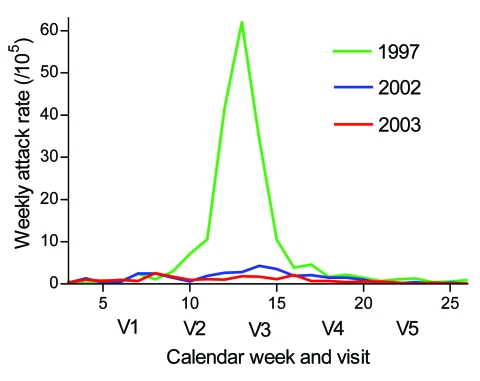
Weekly incidence rates of reported meningitis in the Bobo-Dioulasso region from January through June of 1997, 2002, and 2003. V1, February 3–15 (n = 488); V2, February 25–March 15 (n = 480); V3, March 25–April 12 (n = 465); V4, April 22–May 10 (n = 463); V5, May 27–June 7 (n = 470).

In our study, carriage of the NG:NT ST-192 strain increased from the early phase of the meningitis season to just past its end, as did carriage of *N*. *lactamica* in children. This finding may be an annual phenomenon that is associated with a decreasing meningitis incidence by late April (Figure 4), which would be caused by a reduced risk for infection or disease by virulent meningococci, given the increased carriage prevalence of nongroupable meningococci (*34*). However, our data were from a small sample and only 1 population during 1 meningitis season. A more systematic evaluation by longitudinal carriage studies in several African sites is needed to further explore this hypothesis.

Carriage during this study was dynamic and short-lived compared with other studies in Europe (*15*,*30*,*35*). This finding emphasizes the need for a large sample size in cross-sectional carriage studies and the need for multiple assessment points over short intervals for studies on carriage association with risk factors or immune status. Estimated carriage duration was longer for less virulent nongroupable strains than for NmW135. This could be due to a more accentuated immune response to carriage of encapsulated strains, although serologic evaluation during the same study suggested that the immune response to NmW135 carriage does not occur frequently (*7*).

In the context of hyperendemic NmW135 and NmA disease in Bobo-Dioulasso in 2003, we found a large diversity of phenotypes and genotypes in carried Nm strains (including all strains, except for serogroup A) that caused meningococcal meningitis in this population. NmW135 showed substantial prevalence and high genetic diversity. These features distinguish this serogroup from NmA and indicate that, in combination with poor immune induction by carriage, this serogroup may be a potential epidemic agent. The absence of NmA during this nonepidemic meningitis season and the pronounced dynamics of meningococcal carriage emphasize the need for large samples and a longitudinal design for most carriage studies. By decreasing the risk for infection with a virulent clone, expansion of a nonvirulent clone in carriage toward the end of the meningitis season may be 1 of the mechanisms causing a seasonal decrease in the incidence of meningococcal disease.
